# The impact of pre‐biopsy MRI and additional testing on prostate cancer screening outcomes: A rapid review

**DOI:** 10.1002/bco2.321

**Published:** 2024-01-31

**Authors:** Abel Tesfai, Natalia Norori, Thomas A. Harding, Yui Hang Wong, Matthew David Hobbs

**Affiliations:** ^1^ Prostate Cancer UK London UK

**Keywords:** bpMRI, mpMRI, overdiagnosis, pre‐biopsy, PSA

## Abstract

**Objective:**

This work aims to examine the latest evidence on the impact of pre‐biopsy MRI, in addition to prostate‐specific antigen (PSA) testing, on health outcomes and quality of life.

**Methods:**

We conducted a literature search including PubMed and Cochrane Central Register of Controlled Trials (CENTRAL) databases, with a limited scan of (i) guidelines and (ii) references from trial reports, from January 2005 to 25th January 2023. Two independent reviewers selected randomised controlled trials (RCT) and cohort studies which met our inclusion criteria.

**Results:**

One hundred thirty‐seven articles were identified, and seven trial articles were selected. Trial interventions were as follows: (i) PSA blood test, (ii) additional tests such as pre‐biopsy multiparametric magnetic resonance imaging (mpMRI) and Biparametric MRI (bpMRI), and (iii) MRI targeted biopsy and standard biopsy. Compared with standard biopsy, MRI‐based interventions led to increased detection of clinically significant cancers in three studies and decreased detection of clinically insignificant cancer (Gleason grade 3 + 3) in four studies. However, PROstate Magnetic resonance Imaging Study (PROMIS) and Stockholm3 with MRI (STHLM3‐MRI) studies reported different trends depending on the scenario studied in PROMIS (MRI triage and MRI directed biopsy vs. MRI triage and standard biopsy) and thresholds used in STHLM3‐MRI (≥0·11 and ≥0·15). MRI also helped 8%–49% of men avoid biopsy, in six out of seven studies, but not in STHLM3‐MRI at ≥0.11. Interestingly, the proportion of men who experienced sepsis and UTI was low across studies.

**Conclusion:**

This review found that a combination of approaches, centred on the use of pre‐biopsy MRI, may improve the detection of clinically significant cancers and reduce (i) the diagnosis of clinically insignificant cancers and (ii) unnecessary biopsies, compared with PSA testing and standard biopsy alone. However, the impact of such interventions on longer term outcomes such as prostate cancer‐specific mortality has not yet been assessed.

## 1 INTRODUCTION

The traditional prostate cancer diagnostic pathway used the prostate‐specific antigen (PSA) test and transrectal ultrasonography (TRUS)‐guided 10‐ to 12‐core systematic biopsy. However, the use of PSA to determine diagnostic pathway progression has been linked to overdiagnosis, with one study reporting that 70%–80% of patients with PSA levels >4 ng/mL did not in fact have cancer.^1^ It is also unclear how well PSA levels predict outcomes for low and intermediate‐risk cancers.^2^ Importantly, men who progress through the diagnostic pathway may suffer from physical and psychological issues such as anxiety. More specifically, prostate biopsies have been associated with bleeding, urinary retention issues as well as infectious complications (0.1%–7%) and sepsis (0.3%–3.1%), which can vary depending on the antibiotic prophylaxis regimens implemented. In patients with clinically insignificant cancers, these harms will likely not be associated with any benefit to the patient.^3^, ^4^

The use of magnetic resonance imaging (MRI) has subsequently emerged as a safe and viable tool to detect cancerous lesions within the prostate.^3^, ^5^ MRI images can be taken using two key forms of MRI multiparametric (mp) MRI, using T2‐weighted anatomical imaging, diffusion‐weighted MRI, and dynamic contrast‐enhanced (DCE) MRI and biparametric (bp) MRI, which does not require DCE. Images acquired through bpMRI and mpMRI can be evaluated using diagnostic scoring systems such as Likert and Prostate Imaging‐Reporting and Data System (PI‐RADS) v2.1, to diagnose clinically significant (≥3 + 4; also defined as Gleason ≥7 disease—Gleason grade group 2 or above) and clinically insignificant prostate cancer lesions, that is, 3 + 3; also defined as Gleason 6—Gleason grade group 1.^6^, ^7^, ^8^ Patients identified as having clinically insignificant cancer by MRI can avoid biopsy entirely.

The use of pre‐biopsy MRI was supported by findings from key prospective trials such as PROstate Magnetic resonance Imaging Study (PROMIS) and Prostate Evaluation for Clinically Important Disease: Sampling Using Image‐guidance Or Not (PRECISION), which found that in addition to detecting cancerous lesions within the prostate, MRI could also be used to reduce the number of biopsies performed and increase the number of clinically significant prostate cancer detected.^7^, ^9^ Other studies have also suggested MRI improves the diagnostic pathway and that the introduction of pre‐biopsy MRI as an additional test after PSA screening has reduced the harms of prostate cancer screening.^8^, ^10^ Our previous analysis, using real‐world data, has also suggested benefits of mpMRI in reducing overdiagnosis and associated harms.^11^

In the United Kingdom, MRI is now used after a PSA test to triage men with suspected localised prostate cancer before a biopsy is conducted, as recommended by the National Institute for Health and Care Excellence (NICE) NG131 and Prostate Cancer Risk Management Programme guidelines for asymptomatic individuals. European guidelines also recommend the use of MRI in the diagnostic pathway, but as of writing this review, the United Kingdom remains at the forefront of widespread adoption of MRI into clinical practice.^12^, ^13^, ^14^, ^15^

Our objective in this rapid review is to examine the latest evidence on the role of pre‐biopsy MRI (in addition to PSA) in diagnosing clinically significant cancer, ruling out clinically insignificant cancer and reducing associated harms of PSA and biopsy testing to evaluate the impact on health outcomes and quality of life.

## 2 METHODS

### 2.1 Search strategy

We performed an electronic search using PubMed and Cochrane Central Register of Controlled Trials databases, described in Tables S1–S3.^16^ A limited web search was also conducted for guidelines including the European Association of Urology guideline on diagnostic evaluation, with references from key trials such as PROMIS and PRECISION also checked. Our inclusion criteria encompassed (i) men over 45, (ii) PSA test followed by pre‐biopsy MRI, and/or any other additional testing (intervention), (iii) PSA test only (comparator), and (iv) harms and benefits (outcomes) of prostate cancer screening. The articles retrieved were based on randomised controlled trial (RCT) and cohort studies which were conducted in Europe, the United Kingdom, and Canada (setting) and published in English between 01/01/2005 and 25/01/2023.

### 2.2 Study selection

Retrieved articles were uploaded to the reference system Zotero and screened using systematic tool review Rayyan.^17^ In Rayyan, two independent reviewers conducted a blinded selection process, involving abstract and full text screening, to narrow down articles for review. Conflicts arising from the review process were either resolved between reviewers or reconciled by a third team member.

During the abstract screening process, 124 abstracts were manually screened. Among key decisions made by reviewers were to (i) exclude validation studies that examined biomarkers and polygenic scores and (ii) include three additional articles for full‐text screening. During the full text screening, 15 articles were manually selected. We made the decision not to include a study by Ahdoot et al.^18^ (NCT00102544) on the basis that the whole population of men studied had MRI‐identified lesions. This led to a final selection of seven articles, ready for data extraction.^7^, ^8^, ^9^, ^10^, ^19^, ^20^, ^21^

### 2.3 Data extraction

Data extraction was carried out by three independent reviewers who were randomly assigned to articles using ‘keys’ produced by Zotero. Among extracted data were (i) general information, (ii) method, (iii) participant information, (iv) intervention(s), and (v) outcome(s), as according to Cochrane templates for data extraction.^22^ Following extraction, a single reviewer then re‐examined 30% of the articles assigned to other reviewers to check for completeness and correctness of data.

### 2.4 Risk of bias assessment

Risk of bias assessment was informed by Cochrane's Rapid Review guideline and assessed by a single reviewer, with a second reviewer checking 20% of articles. Reviewers used the main study report, supplementary articles and available protocols, to judge the risk of bias using RoB 2 (one article) and QUADAS‐2 (six articles) tools for RCTs and cohort studies, respectively, across named domains.^23^, ^24^ Five domains were assessed using RoB 2 (version 2019): (i) risk of bias arising from the randomisation process, (ii) risk of bias due to deviations from the intended interventions (effect of assignment to intervention), (iii) risk of bias due to missing outcome data, (iv) risk of bias in the measurement of the outcome, and (v) risk of bias in the selection of the reported result, with an overall risk of bias judged as low, high or some concerns. While for QUADAS‐2, 4 domains were assessed, using tailored signalling questions, specific to the review, along with review‐specific guidance for each domain. The adapted tool was then piloted by two reviewers before use. The four domains were patient selection, index test, reference standard and flow and timing. Moreover, within which signalling questions, risk of bias and concerns regarding applicability were examined. Domains were judged as high, low or some concerns. No exclusions were made based on risk of bias assessment and visual representation of assessments were produced using robvis tool.^25^

## 3 RESULTS

### 3.1 Search results and study characteristics

**FIGURE 1:**
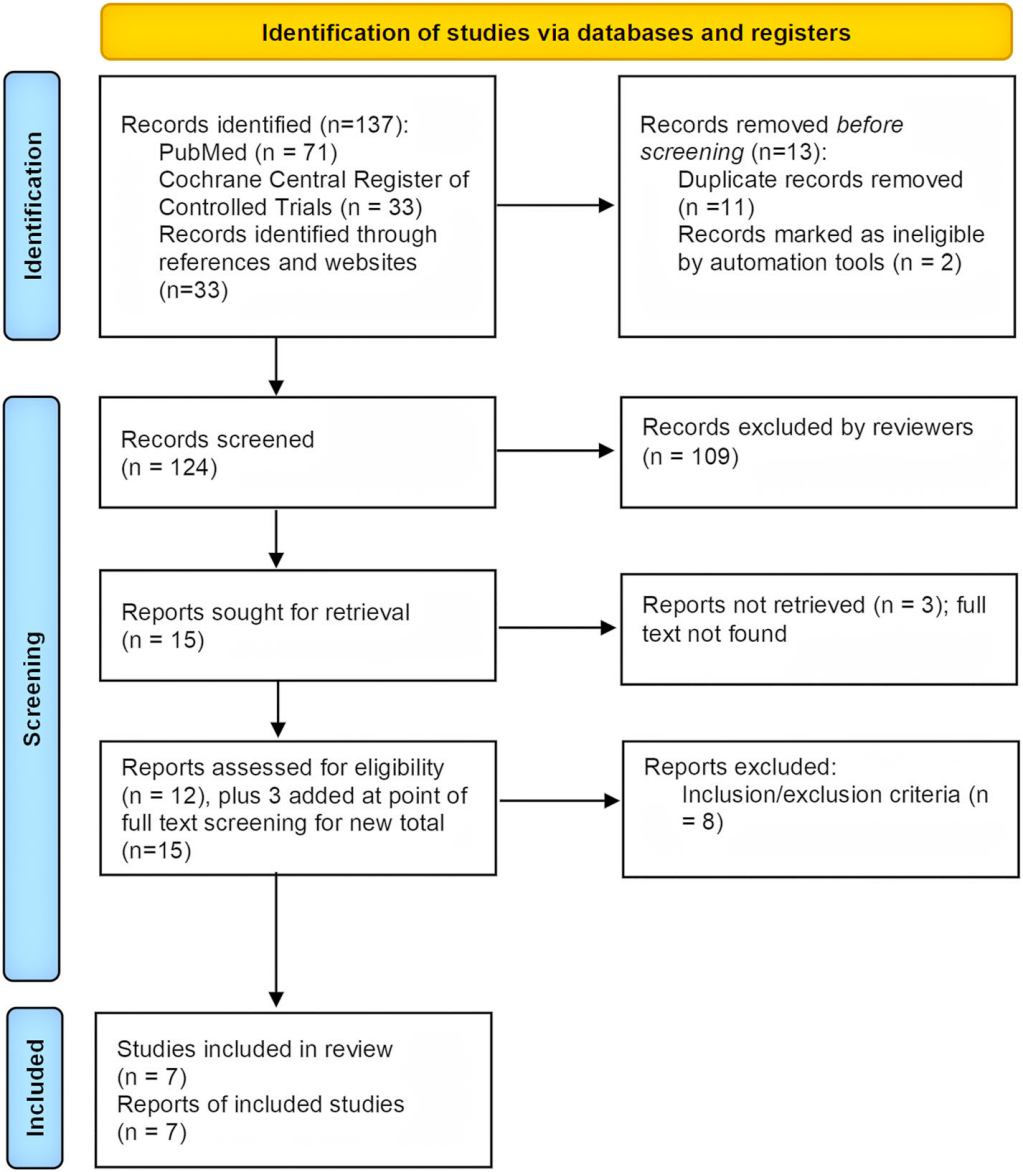
PRISMA flow diagram. Searches were conducted using PubMed, Cochrane Central Register of Controlled Trials, and records identified through references and websites.

One hundred thirty‐seven studies were retrieved using search strategies. From these studies, 124 were screened and seven studies, reporting on seven different prospective trials, were selected for analysis, as described in PRISMA 2020 flow diagram (Figure 1). Full details from trials can be obtained from individual trial numbers displayed in Table 1. Of note, the 4M, Netherlands Trial Register study can be found in the International Clinical Trials Registry Platform search.^25^ The characteristics of the studies included in the analysis are presented in Table 1. Three studies included men who were at least 18 years old and three recruited men aged 50 or older. Only a few studies set an upper age limit as part of their inclusion criteria, requiring participants to be no older than 69, 74, and 75, respectively. One study did not specify any age limits within its inclusion criteria.

**TABLE 1:** Study characteristics. Study details are sourced from relevant articles. All participants were required to be eligible to undergo study protocols, as standard.

				Population inclusion criteria				
Source and trial number	Authors and publication year	Study dates	Study design	Age (years old)	History	Thresholds	Intervention/Comparator	Objective
IP‐1 PROSTAGRAM; ISRCTN43502108	Evans et al. (2021)^10^	October 2018–May 2019	Prospective cohort study	50–69	No prior PSA testing or MRI in last 2 years; no history of UTI or prostatitis in the previous 6 months	NR	All participants underwent PSA test in combination with short bpMRI and ultrasonography (imaging tests were administered irrespective of PSA levels). If any test result was positive, a systematic 12‐core biopsy was performed/PSA test alone	To determine the performance of bpMRI and ultrasonography for prostate cancer screening
PROMIS; ISRCTN16082556	Ahmed et al. (2017)^7^	May 2012–November 2015	Prospective, multicentre, paired cohort confirmatory study	18 years old or over	Clinical suspicion of prostate cancer and advised prostate biopsy	Elevated serum PSA (up to 15 ng/mL) within previous 3 months, suspicious DRE, suspected organ confined stage T2 or lower on rectal examination, or family history	All participants underwent mpMRI followed by targeted biopsy and TRUS biopsy/standard TRUS biopsy	To determine the proportion of men who could (i) safely avoid biopsy and (ii) be correctly identified by mpMRI to have clinically significant prostate cancer. The study also aimed to compare the accuracy of TRUS‐biopsy and mpMRI by examining sensitivity, specificity, positive predictive value, and negative predictive value for clinically significant prostate cancer, with template prostate mapping biopsy used as the reference standard
PRECISION; NCT02380027	Kasivisvanathan et al. (2018)^9^	February 2016–August 2017	Prospective multicentre randomised controlled trial	18 years old or over	No prostate biopsy	PSA of 20 ng/mL per millilitre or less or/and abnormal DRE that did not indicate extracapsular disease in last 3 months	mpMRI and mpMRI‐targeted biopsy if suspicious MRI results/standard TRUS biopsy	To compare the performance of MRI‐targeted biopsy with standard TRUS biopsy
BIDOC; NCT02584179	Boesen et al. (2018)^8^	November 2015–June 2017	Prospective single centre, paired cohort study	18–85	N/A	PSA ≥4 ng/mL and/or abnormal DRE	All patients underwent pre biopsy mpMRI, systematic TRUS biopsy, and mpMRI‐targeted biopsy if suspicious MRI results/standard TRUS biopsy	To assess the diagnostic accuracy of bpMRI for detecting clinically significant prostate cancer in biopsy‐naive men, and to determine whether bpMRI can be used as a simpler and faster alternative to detecting prostate cancer.
4M; NTR5555	Van der Leest et al. (2019)^21^	February 2015–February 2018	Prospective, multicentre, powered, comparative effectiveness study	50–75	No history of (i) prostate cancer biopsy and treatment, (ii) contraindications for MRI, (iii) taking medication that affects level of serum PSA, (iv) symptoms of UTI, and (v) invasive treatment for benign hyperplasia	PSA of ≥3 ng/mL	All patients underwent pre biopsy mpMRI followed by systematic TRUS biopsy, and MRI‐targeted biopsy if suspicious MRI results prior to TRUS guided biopsy/standard TRUS biopsy	To compare the effectiveness of the MRI pathway with the TRUS guided biopsy pathway in detecting clinically significant prostate cancer.
STHLM3‐MRI; NCT03377881	Nordström et al. (2021)^19^	February 2018–March 2020	Prospective, population‐based, randomised, open‐label (no blinding), non‐inferiority trial	50–74	No contraindications to use of MRI	Elevated risk of prostate cancer based on PSA or STHLM3 score: PSA score ≥1.5 ng/mL, and PSA of ≥3 ng/mL or STHLM3 of ≥0·11	bpMRI followed by MRI‐targeted and systematic TRUS biopsy if suspicious MRI results/standard TRUS biopsy	To assess whether a risk prediction model (the STHLM3 test) can improve the selection of men undergoing MRI as a part of a population‐based screening strategy for prostate cancer
MRI‐FIRST; NCT02485379	Rouvière et al. (2019)^20^	July 2015–August 2016	Prospective, multicentre, paired diagnostic study	18–75 Men aged between 18 and 75 years	No history of (i) androgen deprivation therapy, (ii) hip prosthesis, (iii) pelvic radiotherapy, or (iv) diagnosis after transurethral resection of the prostate.	Suspicion of prostate cancer; PSA of ≤20 ng/mL, with DRE that did not suggest extracapsular (T3) disease	mpMRI followed by MRI guided biopsy if suspicious MRI results. All patients underwent both MRI‐targeted and systematic biopsies at a single institution/standard TRUS biopsy	To investigate whether mpMRI improves the detection of clinically significant prostate cancer in biopsy naive patients.

Abbreviations: 4M, Met Prostaat MRI Meer Mans; BIDOC, Biparametric MRI for Detection of Prostate Cancer; bpMRI, biparametric Magnetic Resonance Imaging; DRE, digital rectal examination; IP‐1 PROSTAGRAM, Imperial Prostate 1 Prostate Cancer Screening Trial Using Imaging; ISRCTN, International Standard Randomised Controlled Trial Number; mpMRI, multiparametric Magnetic Resonance Imaging; MRI, magnetic resonance imaging; MRI‐FIRST, Assessment of Prostate Magnetic Resonance Imaging Before Prostate Biopsies; NCT, National Clinical Trial Number; PRECISION, PRostate Evaluation for Clinically Important Disease: Sampling Using Image‐guidance Or Not?; PROMIS, PROstate Magnetic resonance Imaging Study; PSA, prostate‐specific antigen; STHLM3‐MRI, Stockholm3 with Magnetic Resonance Imaging; T, size of tumour (stage); TRUS, transrectal ultrasonography; UTI, urinary tract infection.

The studies included showed substantial heterogeneity in terms of population characteristics, type of pre‐biopsy MRI assessed, PSA thresholds, MRI scoring, and definition of clinically significant prostate cancer. Three of the studies, namely, PROMIS, PRECISION, and IP1‐PROSTAGRAM, were conducted in the United Kingdom, while four studies, namely, Biparametric Magnetic Resonance Imaging for Detection of Prostate Cancer (BIDOC), Stockholm3 with Magnetic Resonance Imaging (STHLM3‐MRI), the 4M study, and Assessment of Prostate Magnetic Resonance Imaging Before Prostate Biopsies (MRI‐FIRST), were conducted in European countries.

### 3.2 Quality assessment

**FIGURE 2:**
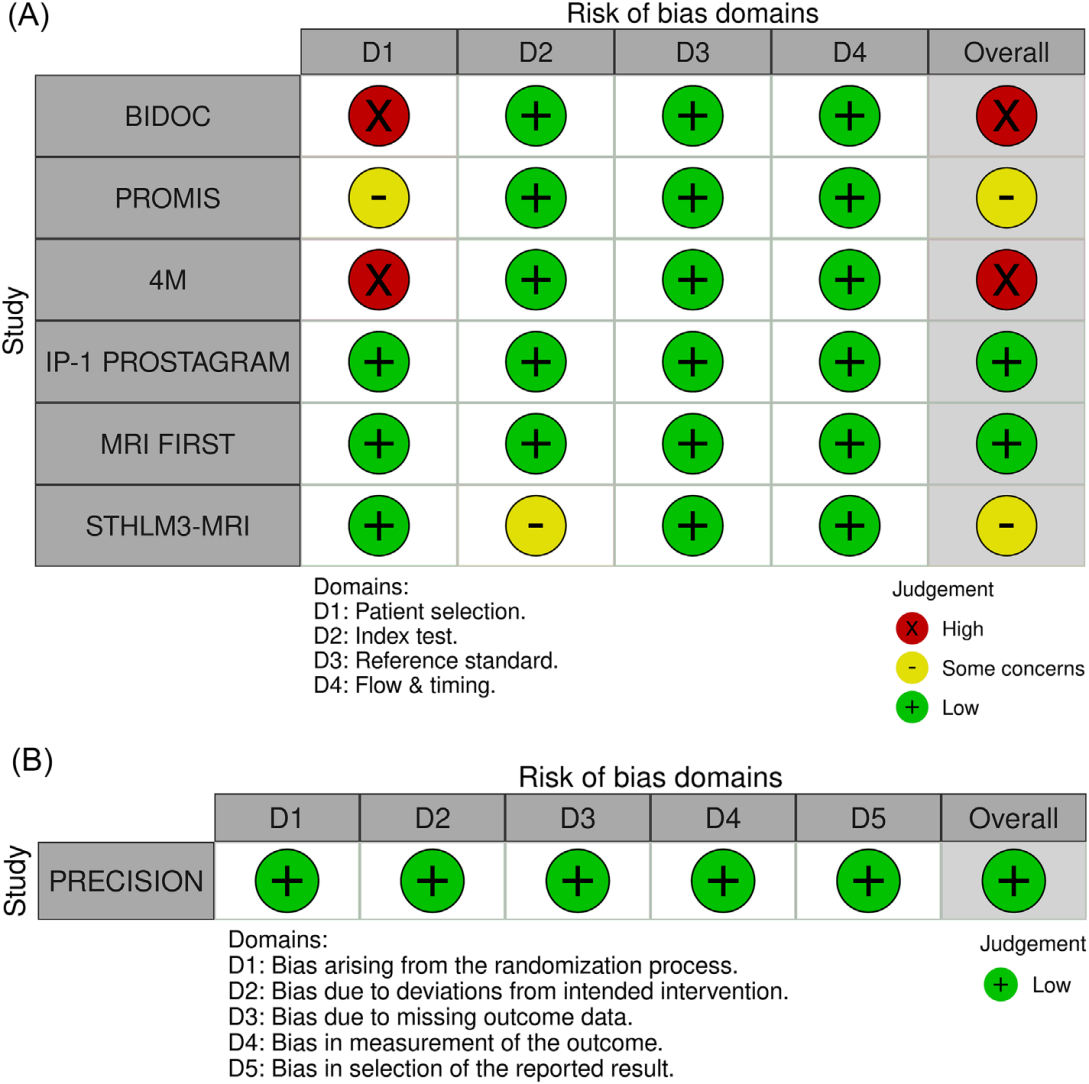
(A,B) Risk of bias summaries for diagnostic accuracy studies; QUADAS‐2 (A) and randomised controlled trials; RoB2 (B). QUADAS‐2 tool was used to assess risk of bias and applicability concerns in diagnostic accuracy studies. All studies had low risk of bias rating but had different applicability concerns; therefore applicability concerns were selected for display in Figure 2A. Abbreviations: 4M, Met Prostaat MRI Meer Mans; BIDOC, Biparametric MRI for Detection of Prostate Cancer; IP‐1 PROSTAGRAM, Imperial Prostate 1 Prostate Cancer Screening Trial Using Imaging; MRI‐FIRST, Assessment of Prostate Magnetic Resonance Imaging Before Prostate Biopsies; PRECISION, PRostate Evaluation for Clinically Important Disease: Sampling Using Image‐guidance Or Not?; PROMIS, PROstate Magnetic resonance Imaging Study; STHLM3‐MRI, Stockholm3 with Magnetic Resonance Imaging.

Using QUADAS‐2, six studies were rated as having low risk of bias overall. However, there were differences in concerns regarding applicability with IP‐1 PROSTAGRAM and MRI‐FIRST judged to have low concern regarding applicability, while (i) PROMIS and STHLM3‐MRI and (ii) BIDOC and 4M were judged as having some and high concerns regarding applicability, respectively (Figure 2). The only RCT trial (PRECISION) examined in this review was judged as low risk overall, when using RoB 2 tool of assessment.

### 3.3 Population characteristics

A total of 5674 participants were included in the analysis. The number of participants included varied considerably between studies from 251 to 2293 patients with median ages between 63 and 67 years (Table 2).^26^ Three studies included biopsy naive men (PRECISION, IP‐1 PROSTAGRAM, and BIDOC), and the rest did not specify. Six studies included men with a clinical suspicion for prostate cancer determined by either a raised PSA level, STHLM3 score, or abnormal DRE.

**TABLE 2:** Population characteristics. Study details are sourced from relevant articles for index (MRI) test. For BIDOC, 4M, STHLM3‐MRI study, and MRI‐FIRST, the average age of men was converted from median and IQR to mean and SD, based on Hozo et al.^26^ to enable comparison.

Source	Number of men included in analysis	Number of men with prostate cancer (% of total)	Number of men with csPCa (% of total)	Age; mean (SD)	Prostate cancer status
IP‐1 PROSTAGRAM^10^	408	37 (9%)	17 (4.2%)	NR	No suspected prostate cancer
PROMIS^7^	576	408 (70.8%)	230 (39.9%)	63.4 years (±7.6)	Clinical suspicion of prostate cancer
PRECISION^9^	500	237 (47.4%)	159 (31.8%)	Intervention group, *n* = 252, 64.4 years (±7.5) Control group, *n* = 248, 64.5 years (± 8.0)	Clinical suspicion of prostate cancer
BIDOC^8^	1020	655 (64.2%)	404 (39.6%)	66.5 years (± 2.9)	Clinical suspicion of prostate cancer
4M^21^	626	334 (53.3%)	190 (30.4%)	64.3 years (±2.6)	Clinical suspicion of prostate cancer
STHLM3‐MRI^19^	2293	993 (43.3%)	403 (17.6%)	Intervention group, *n* = 1372, 66.0 years (±2.9) Control group, *n* = 921, 66.5 years (±2.9).	Clinical suspicion of prostate cancer
MRI‐FIRST^20^	251	150 (59.7%)	94 (37.5%)	63.8 years (±2.6)	Clinical suspicion of prostate cancer

Abbreviations: 4M, Met Prostaat MRI Meer Mans; BIDOC, Biparametric MRI for Detection of Prostate Cancer; csPCa, clinically significant prostate cancer; IP‐1 PROSTAGRAM, Imperial Prostate 1 Prostate Cancer Screening Trial Using Imaging; MRI‐FIRST, Assessment of Prostate Magnetic Resonance Imaging Before Prostate Biopsies; NR, not reported; PRECISION, PRostate Evaluation for Clinically Important Disease: Sampling Using Image‐guidance Or Not?; PROMIS, PROstate Magnetic resonance Imaging Study; SD, standard deviation; STHLM3‐MRI, Stockholm3 with Magnetic Resonance Imaging.

Of the seven studies, three evaluated the effectiveness of mpMRI followed by MRI‐targeted biopsy in men with suspicious MRI results, and two studies assessed the performance of bpMRI followed by MRI‐targeted biopsy, in men with suspicious MRI results, as opposed to using standard TRUS biopsy alone. One study compared the performance of PSA testing, bpMRI, and ultrasonography as screening tests for prostate cancer (IP‐1 PROSTAGRAM).

A Likert, PI‐RADS v2, or modified PI‐RADS v2 score of 3–5 was perceived as suspicious across studies. Thresholds for clinically significant prostate cancer were reported by all studies. Five studies defined clinically significant prostate cancer as Gleason grade ≥3 + 4 and two studies as Gleason grade 4 + 3. Only the primary definitions of clinically significant prostate cancers were included in our analysis. The key outcomes measured, thresholds, and definitions used are presented in Table 3.

**TABLE 3:** Outcomes measured and key study definitions, sourced from study articles.

Source	Outcomes measured	csPCa definition	Raised PSA threshold	Suspicious MRI threshold (scoring system)	Type of MRI (magnet strength)
IP‐1 PROSTAGRAM^10^	Detection of clinically significant and insignificant prostate cancer by biopsy after bpMRI, ultrasonography, and PSA, adverse events after biopsy	Gleason score ≥3 + 4	≥3 ng/mL	3 to 5 and 4 to 5 (PI‐RADS v2)	BP (1.5‐T or 3.0‐T)
PROMIS^7^	Prostate cancer prevalence, detection of clinically significant and insignificant prostate cancer, biopsies avoided, diagnostic accuracy of mpMRI and TRUS biopsy, adverse events after biopsy	Primary definition: Gleason score ≥4 + 3 or cancer core length ≥6 mm	Up to 15 ng/mL	3 to 5 (Likert)	MP (1.5‐T)
PRECISION^9^	Prostate cancer prevalence, detection of clinically significant and insignificant prostate cancer, biopsies avoided, quality of life, adverse events after biopsy	Gleason score ≥3 + 4	Up to 20 ng/mL	3 to 5 (PI‐RADS v2)	MP (1.5‐T or 3.0‐T)
BIDOC^8^	Prostate cancer prevalence, detection of clinically significant and insignificant prostate cancer, biopsies avoided diagnostic accuracy bpMRI	Primary definition: Gleason score of ≥4 + 3 or maximum cancer‐core length greater than 50% with a Gleason score of 3 + 4.	≥4 ng/mL	3 to 5 (modified PI‐RADS v2)	BP (3.0‐T)
4M^21^	Prostate cancer prevalence, detection of clinically significant and insignificant prostate cancer, biopsies avoided, adverse events after biopsy	Gleason score ≥3 + 4	≥3 ng/mL	3 to 5 (PI‐RADS v2)	MP (3.0‐T)
STHLM3‐MRI^19^	Prostate cancer prevalence, detection of clinically significant and insignificant prostate cancer, biopsies avoided	Gleason score ≥3 + 4	≥3 ng/mL (comparator to STHLM3 scores)	3 to 5 (modified PI‐RADS v2)	BP (1.5‐T or 3.0‐T)
MRI‐FIRST^20^	Prostate cancer prevalence, detection of clinically significant and insignificant prostate cancer, biopsies avoided	ISUP classification (2014): grade group 2 or higher tumours, i.e., Gleason score ≥3 + 4	Up to 20 ng/mL	3 to 5 (Likert and PI‐RADS v2)	MP (1·5‐T or 3.0‐T)

Abbreviations: 4M, Met Prostaat MRI Meer Mans; BIDOC, Biparametric MRI for Detection of Prostate Cancer; bpMRI, biparametric Magnetic Resonance Imaging; csPCa, clinically significant prostate cancer; IQR; interquartile range; ISUP, International Society of Urological Pathology; IP‐1 PROSTAGRAM, Imperial Prostate 1 Prostate Cancer Screening Trial Using Imaging; MRI‐FIRST, Assessment of Prostate Magnetic Resonance Imaging Before Prostate Biopsies; mpMRI, multiparametric Magnetic Resonance Imaging; NR, not reported; PCa; prostate cancer; PI‐RADS, prostate imaging—reporting and data system; PRECISION, PRostate Evaluation for Clinically Important Disease: Sampling Using Image‐guidance Or Not?; PROMIS, PROstate Magnetic resonance Imaging Study; STHLM3‐MRI, Stockholm3 with Magnetic Resonance Imaging; T, tesla.

### 3.4 Prostate cancer prevalence

The prevalence of prostate cancer varied significantly across the six studies that included men with a clinical suspicion of the disease, ranging from 43.3% to 70.8%. In the IP‐1 PROSTAGRAM study, which did not restrict the inclusion criteria to men with a clinical suspicion of prostate cancer, the prevalence of prostate cancer was much lower at 9%.

### 3.5 Detection of clinically significant prostate cancer

Three primary definitions of clinically significant prostate cancer were used. Five studies, namely, IP‐1 PROSTAGRAM, PRECISION, the 4M study, STHLM3‐MRI, and MRI‐FIRST, defined clinically significant prostate cancer as Gleason grade ≥3 + 4, whereas BIDOC defined significance as Gleason score of ≥4 + 3 or maximum cancer‐core length greater than 50% with a Gleason score of 3 + 4. In PROMIS, clinically significant cancer was primarily defined as Gleason grade ≥4 + 3 at any core length, or a maximum cancerous core length of ≥6 mm at biopsy.

Most studies that used the definition Gleason grade ≥3 + 4, reported an increase in the detection of clinically significant prostate cancer by MRI triage when compared with standard TRUS biopsy (Table 4). Increases in clinically significant prostate cancer ranged from 2% in 4M study to 15% in STHLM3‐MRI study (at ≥0.11 threshold). Also, both the MRI‐FIRST study and STHLM3‐MRI (at ≥0.15 threshold) reported no difference in the proportion of clinically significant prostate cancer detected by MRI triage and standard TRUS biopsy.

**TABLE 4:** Key outcomes across studies that compared MRI triage (bp or mp) followed by MRI‐targeted biopsy with standard TRUS biopsy alone (relative values). IP‐1 PROSTAGRAM has not been included in this table as it compares MRI and ultrasonography to PSA test alone.

Source	% detection of csPCa	% detection of cisPCa	% who avoid biopsy
PROMIS best‐case scenario^7^	18% increase	5% increase	27%
PROMIS worst‐case scenario^7^	1% decrease	5% decrease	27%
PRECISION^9^	12% increase	13% decrease	28%
BIDOC^8^	11% increase	40% decrease	30%
4M^21^	2% increase	11% decrease	49%
STHLM3‐MRI;STHLM3 ≥0·11 ^19^	15% increase	22% increase	None, 18% more biopsies administered
STHLM3‐MRI;STHLM3 ≥0·15 ^19^	0% increase (no change)	17% decrease	8%
MRI‐FIRST^20^	No difference	25% decrease	21%

*Note*: PROMIS best‐case scenario: mpMRI triage followed by MRI directed TRUS‐biopsy; PROMIS worst‐case scenario: mpMRI triage followed by standard non‐directed TRUS biopsy.

Abbreviations: 4M, Met Prostaat MRI Meer Mans; BIDOC, Biparametric MRI for Detection of Prostate Cancer; bpMRI, biparametric Magnetic Resonance Imaging; cisPCa, clinically insignificant prostate cancer; csPCa, clinically significant prostate cancer; MRI‐FIRST, Assessment of Prostate Magnetic Resonance Imaging Before Prostate Biopsies; PRECISION, PRostate Evaluation for Clinically Important Disease: Sampling Using Image‐guidance Or Not?; PROMIS, PROstate Magnetic resonance Imaging Study; PSA, prostate‐specific antigen; STHLM3‐MRI, STHLM3 with Magnetic Resonance Imaging.

Although PROMIS and BIDOC used different definitions for clinically significant prostate cancer, they also report a greater number of clinically significant cases detected by MRI triage, 11% in BIDOC and 18% in PROMIS under the best‐case scenario, that is, mpMRI triage followed by mpMRI directed TRUS biopsy. However, in the PROMIS study's worst‐case scenario, defined as mpMRI triage followed by standard nondirected TRUS biopsy, there was a 1% decrease in the diagnosis of clinically significant disease when compared with standard TRUS biopsy alone.

Furthermore, MRI‐FIRST, 4M, and BIDOC evaluated the performance of combined biopsies (MRI‐targeted plus standard TRUS) and found that a combined approach enhanced the detection of significant prostate cancer. The IP‐1 PROSTAGRAM study compared MRI to PSA test alone and found that MRI detected twice as many clinically significant cases.

### 3.6 Detection of clinically insignificant prostate cancer

Among studies that defined clinically insignificant prostate cancer as Gleason grade 3 + 3, four reported that MRI triage led to a reduction in the number of clinically insignificant cases ranging between 13% decrease in PRECISION study to 25% in MRI‐FIRST study (Table 4).

The BIDOC study, which defined clinically insignificant cancer as Gleason grade 3 + 3 and 3 + 4 with maximum cancerous core length of ≤50%, found that MRI triage detected 40% fewer clinically insignificant cases than when using standard biopsies. In contrast, the PROMIS trial found that MRI triage followed by MRI directed TRUS biopsy may lead to a 5% increase in overdiagnosis of clinically insignificant prostate cancer (Gleason grade ≥4 + 3), under the best‐case scenario.

### 3.7 Proportion of men who are ruled out of biopsy

Five studies have demonstrated that using MRI triage before biopsy can rule out between 21% and 49% of men from undergoing biopsy. By only performing biopsies on men with suspicious MRI results, PROMIS, PRECISION, and BIDOC avoided 27%, 28%, and 30% of unnecessary biopsies, respectively. Similarly, the 4M study found that MRI triage avoided 49% of unnecessary biopsies, and the MRI‐FIRST study found that MRI triage avoided 21% of unnecessary biopsies (Table 4). However, in the STHLM3‐MRI study, a STHLM3 score of ≥0·11 resulted in an 18% increase in the number of biopsies, while a score of ≥0·15 led to an 8% reduction in the number of men advised to undergo biopsy, after MRI triage, when compared with the PSA test alone. The use of STHLM3 ≥0·11 threshold also led to the detection of more clinically significant and insignificant cancers while STHLM3 ≥0·15 was associated with a reduction in the detection of clinically insignificant cancers.

### 3.8 Sepsis and urinary tract infection (UTI) after biopsy

The proportion of men who experienced sepsis and UTI was low across studies. However, there were some variations in the rates reported. In the PROMIS trial, only 1% of participants experienced sepsis and 6% developed UTI after a combined biopsy procedure. In the PRECISION trial, 0.4% experienced sepsis after MRI‐targeted biopsy and 1.6% after standard TRUS biopsy. In PRECISION, 30 days post intervention, 2.4% reported experiencing UTI after MRI‐targeted biopsy and 1% after standard TRUS biopsy. Similarly, the 4M study reported that 3% of men experienced sepsis after a combined biopsy procedure. STHLM3‐MRI reported that 1.8% of men were prescribed antibiotics for infection in the MRI triage group and 4.4% in the standard TRUS biopsy group.

### 3.9 Other outcomes

We were unable to draw conclusions on the impact of the MRI pathway on overtreatment psychological effects, incidence of metastatic prostate cancer, all‐cause mortality, and prostate cancer‐specific mortality based on the studies included in the analysis. This indicates limited amount of information on outcome, in line with recent systematic reviews.^27^

## 4 DISCUSSION

In the United Kingdom, results from key trials have helped embed the use of pre‐biopsy MRI and MRI targeted biopsies to the modern diagnostic pathway.^7^, ^9^, ^12^ This review shows that the application of MRI for men suspected of having prostate cancer can help reduce overdiagnosis of clinically insignificant cancers and increase the detection of clinically significant cancers. However, the impact of MRI in screening remains unclear.

When using MRI, the detection of clinically significant cancers was found to be increased, across most studies (2%–18% increase), when compared with standard biopsy (Table 4), while the detection of clinically insignificant cancers was found to be reduced (5%–40% decrease). However, it was interesting to note that in PROMIS, the use of MRI to detect cancer and target biopsies (best‐case scenario) resulted in a 5% increase in the detection of clinically insignificant prostate cancer, whereas under the worst‐case scenario, where mpMRI triage was followed by standard nondirected TRUS biopsy, no such increase was found. Moreover, it is important to note that the main finding of the MRI‐FIRST study was that a combined approach using MRI targeted biopsy and systematic biopsy would increase the detection of clinically significant prostate cancer compared with when using either MRI targeted biopsy or systematic biopsy alone. This review also found that across most studies the percentage of men who avoided biopsies, after pre‐biopsy MRI, ranged from 21% to 49% (Table 4). This finding was also supported by other reviews, which found that MRI could be used to avoid biopsies.^28^, ^29^, ^30^

There was limited data on patient outcomes and quality of life. Where data were available, these were based on short‐term biopsy‐related measurements including rates of infection and sepsis. More specifically, the following studies—PROMIS, PRECISION, and 4M—linked the use of MRI with reduced rates of infection and sepsis, in concordance with past reviews.^30^ The STHLM3‐MRI study also suggested reduced infection via proxy data, namely, a reduction in number of men prescribed antibiotics, when compared with TRUS biopsy group. The exact reasons for reduced rates of infection were not clear but may be due to a combination of reasons, including reduced number of biopsy cores, when using MRI‐targeted biopsy, and application of specific prophylaxis antibiotic regimen.^31^

Furthermore, examined studies did not report longer term outcomes such as incidence of metastatic cancer and cancer mortality. Participants in PROMIS have consented to the use of central registries data for follow‐up studies. Moreover, neither IP‐1 PROSTAGRAM nor STHLM3‐MRI reported on these longer term outcomes or provided screening‐specific measurements such as number needed to invite to screen and number needed to diagnose prostate cancer to avoid one prostate cancer death.

Past reports have indicated that MRI can be used to detect and rule out cancer as well as avoid biopsy.^11^, ^28^ In the United Kingdom, NICE guidelines (NG131) recommend the use of pre‐biopsy MRI as an initial investigation for people with suspected localised prostate cancer.^14^ Moreover, an examination of real‐world evidence data, relating to an Information Act request conducted in 2019, has highlighted the proportion of areas that offer mpMRI was more than 90% across the United Kingdom, an increase from previously published work^15^ demonstrating widespread accessibility of MRI, despite some regional variation (Table S4).

This review has some key limitations. First, we did not report and compare measures of (i) test performance such as sensitivity and specificity and (ii) diagnostic effectiveness such as negative and positive predicative values across studied interventions and controls, although it is well known that both bpMRI and mpMRI have a high negative predicative value and sensitivity for the detection of clinically significant prostate cancer. Previous reports have suggested this translates to reduction in harms such as overdiagnosis, caused by PSA testing.^7^, ^8^ Second, there was a lack of follow‐up studies detailing long‐term patient outcomes following MRI, thereby posing a considerable risk that this review could overestimate the benefits of MRI and underreport the long‐term harms of the PSA followed by MRI pathway. Of relevance, one study has measured the impact of MRI on prognostic model performance, including NICE‐Cambridge Prognostic Group system, in lieu of survival data^32^.

However, it is important to note that some of these harms have also been reduced for reasons other than the pathway change investigated here such as the use of transperineal biopsy, which is associated with reduced prevalence of sepsis (0.1%) when compared with transrectal (0.8%) biopsy.^33^, ^34^

Furthermore, there have been several developments in the field, which may enhance the diagnostic pathway that did not meet our inclusion criteria.^3^ One relatively recent development, reported in this review, was the use of STHLM3 test as part of the diagnostic pathway. STHLM3 test scores are based on a combination of a selection of markers, including genetic polymorphisms, plasma protein biomarkers (PSA; total and free, glandular kallikrein, microphage inhibitory cytokine‐1 and microseminoprotein beta), and patient characteristics. Previously, the use of STHLM3 alongside the PSA test has been suggested to potentially reduce the number of cancers missed due to normal levels of PSA and also reduce the number of biopsies by as much as 44%.^35^, ^36^, ^37^ The STHLM3‐MRI trial study article, examined in this review, assessed how men with an elevated prostate cancer risk, based on either a PSA of 3 ng/mL or STHLM3 score of ≥0.11 or ≥0.15, benefit from additional intervention by bpMRI and targeted biopsy. Interestingly, the use of ≥0·11 as a threshold was associated with an increase in both the detection of clinically significant and insignificant cancers as well as an increase in the percentage of biopsies administered, suggesting low specificity (Table 4), whereas the use of STHLM3 score ≥0.15 was associated with a relative decrease the detection of clinically insignificant cancers when compared with standard group (systematic biopsy); notably, this review also found a modest decrease in percentage of men (8%) who underwent biopsy at this threshold.^19^ Importantly, the relative cost of the STHLM3 test compared with PSA and the unknown long‐term impact on prostate cancer‐specific mortality reduce the feasibility of widespread adoption of this technology in the United Kingdom and elsewhere. Furthermore, study investigators from a recent IP‐1 PROSTAGRAM statistical report suggested that the use of a combination of PSA ≥1 ng/mL and MRI score ≥4 thresholds, in the first round of future screening programmes, may facilitate the detection of grade group ≥2 cancers, while reducing biopsy referrals, suggesting multiple viable routes to reducing harms associated with overdiagnosis.^38^

To our knowledge, this is the first review to report on improvements in both diagnostic accuracy and reduction of harm due to innovations in the early section of the diagnostic pathway. Studies investigating the use of MRI reported reduced diagnosis of clinically insignificant cancers, reduced unnecessary biopsies, and improved detection of clinically significant prostate cancers. However, we did not find evidence on the impact of pre‐biopsy MRI triage on longer term outcomes such as incidence of metastatic disease and prostate cancer‐specific mortality. Thus, further research is required to determine if the harms and benefits of PSA‐based screening have changed since the introduction of MRI. Ideally, these investigations will involve the development of high‐quality RCTs, which seek to examine both short‐term and longer term outcomes including prostate cancer incidence, rates of biopsy and infection, prostate‐specific mortality, number needed to invite to screen, and number needed to diagnose prostate cancer to avoid one prostate cancer death, overall mortality as well as quality of life outcomes.^39^ Future studies, based on real‐world evidence, may also provide important insights on the impact of pre‐biopsy MRI as part of the modern diagnostic pathway.^11^

## AUTHOR CONTRIBUTIONS

The authors named in the author section contributed to the creation of this manuscript.

## CONFLICT OF INTEREST STATEMENT

The authors in this review declare no conflict of interest.

## Supporting information


**Table S1.** Study protocol and inclusion/exclusion criteria.
**Table S2.** Search terms and combinations used in PubMed. The following selection filters to final search line: English language, clinical trials and randomised controlled trial studies, published between 01/01/2005 and 25/01/2023.
**Table S3.** Search terms and combinations used in Cochrane Central Register of Controlled Trials. The following selection filters to final search line: English language, clinical trials and randomised controlled trial studies, published between 01/01/2005 and 25/01/2023.
**Table S4.** Proportion of areas providing mpMRI, 2019 Freedom of Information results.
