# Supplementary material for: The impact of pre‐biopsy MRI and additional testing on prostate cancer screening outcomes: A rapid review
Source: BJUI Compass. 2024 Jan 31;5(4):426–38. doi: 10.1002/bco2.321 (PMC11019254; doi:10.1002/bco2.321)
Supplement: Supplementary file 1 — Table S1. Study protocol and inclusion/exclusion criteria. Table S2. Search terms and combinations used in PubMed. The following selection filters to final search line: English language, clinical trials and randomised controlled trial studies, published between 01/01/2005 and 25/01/2023. Table S3. Search terms and combinations used in Cochrane Central Register of Controlled Trials. The following selection filters to final search line: English language, clinical trials and randomised controlled trial studies, published between 01/01/2005 and 25/01/2023. Table S4. Proportion of areas providing mpMRI, 2019 Freedom of Information results. [file BCO2-5-426-s001.docx]

**Supplementary Tables**

***Supplementary Table 1.*** *Study protocol and inclusion/exclusion criteria.*

| **Study Protocol** | **Details** | |
| --- | --- | --- |
| **Review Question** | Have the benefits and harms of PSA-based prostate cancer screening changed since the introduction of pre-biopsy MRI and additional testing into the prostate cancer pathway? | |
| **Type of study to be reviewed** | Randomised controlled trials and cohort studies | |
| **Method of review** | Rapid review | |
| **Main condition of interest** | Prostate cancer | |
| **Aim of study** | To determine whether MRI and other tests have favourably shifted the harms and benefits of PSA-based testing on health outcomes and quality of life | |
| **Date** | 01/01/2005-25/01/2023 | |
| **Language** | English | |
| **Country** | Europe, the United Kingdom and Canada. The country selection was informed by national and international guidelines on the use of pre-biopsy MRI on asymptomatic men | |
| **Information sources and databases** | Relevant articles were retrieved from PubMed and Cochrane Central Register of Controlled Trials via Wiley. A limited search of grey literature was also conducted; this included scanning references from European Association of Urology diagnostic evaluation guideline and key trials such as PROMIS and PRECISION | |
| **Screening process** | The Rayyan systematic review tool will be used by two independent reviewers to conduct abstract and full-text screening. The Rayyan systematic tool will be used to enable i) the removal of duplicate articles found from the use of different sources and search strategies, ii) enable a blinded selection process using separate login systems, and iii) enable the use of both manual and automated screening and selection process. Conflicts between reviewers, found after unblinding decisions, will be resolved through a third team member | |
| **Study records and assignment of articles for data extraction** | Zotero reference management system will be used to store references of articles selected via Rayyan, using BibTeX format. Zotero will also be used to generate ‘keys’ (codes), to facilitate the random assignment of articles to reviewers ahead of data extraction | |
| **Data extraction** | The data extraction form will be developed and tested using Excel. The data extraction process will gather information from relevant materials including the main study report, supplementary papers, and available protocols:   - general information - methods - participants - intervention - outcome - others - funding sources, conflict of interest and study conclusions | |
| **Risk of bias assessment tools** | Randomised controlled trials and cohort studies. The following quality assessment tools were used: risk of bias for randomised controlled trials and QUADAS2 for diagnostic accuracy studies | |
| **Review team details** | - Abel Tesfai: [abel.tesfai@prostatecanceruk.org](mailto:abel.tesfai@prostatecanceruk.org) - Natalia Norori: [natalia.norori@prostatecanceruk.org](mailto:natalia.norori@prostatecanceruk.org) - Thomas A Harding: [tom.harding@bristol.ac.uk](mailto:tom.harding@bristol.ac.uk) - Yui Hang Wong: [harris.wong@prostatecanceruk.org](mailto:harris.wong@prostatecanceruk.org) - Matthew David Hobbs: [matthew.hobbs@prostatecanceruk.org](mailto:matthew.hobbs@prostatecanceruk.org) | |
| **Review team organisational affiliation** | Prostate Cancer UK | |
| **Population, Intervention, Comparator and Outcome (PICO)** | **Inclusion criteria** | **Exclusion criteria** |
| **Population** | Men over 45 | Men who have been previously diagnosed with prostate cancer. Men under 45 |
| **Intervention(s)** | PSA test followed by follow-up testing (pre biopsy MRI) and any other additional testing | PSA test only |
| **Comparator(s)** | PSA test | - |
| **Outcome(s)** | - Benefits: reduction in prostate cancer-specific mortality, reduction in all-cause mortality, reduction in the incidence of aggressive/metastatic disease, improvement in quality of life, and psychological effects. - Harms: overtreatment and treatment specific harmful effects, overdiagnosis, complications due to biopsy (UTI, Sepsis, Infection), psychological effects | - |

***Supplementary Table 2.*** *Search terms and combinations used in PubMed.* The following selection filters to final search line: English language, clinical trials and randomised controlled trial studies, published between 01/01/2005 and 25/01/2023.

| #1 | Prostatic neoplasms OR prostate cancer |
| --- | --- |
| #2 | Middle Aged OR Aged |
| #3 | #1 AND #2 |
| #4 | PSA or Prostate-Specific Antigen/blood* |
| #5 | Mass screening/methods* OR Early Detection of Cancer |
| #6 | Magnetic Resonance Imaging /methods OR Magnetic Resonance Imaging* OR Biparametric Magnetic Resonance Imaging OR Multiparametric Magnetic Resonance Imaging |
| #7 | Image-Guided Biopsy OR pre-biopsy MRI OR (pre-biopsy MRI) - |
| #8 | Risk Assessment* OR Risk Stratification OR (Cambridge Prognostic group* OR CPG) OR (D'Amico OR Damico) |
| #9 | #4 AND (#5 OR #6 OR #7 OR #8) |
| #10 | PSA OR Prostate-Specific Antigen / blood* |
| #11 | Magnetic Resonance Imaging / methods OR Magnetic Resonance Imaging* OR Biparametric Magnetic Resonance Imaging OR Multiparametric Magnetic Resonance Imaging |
| #12 | Image-Guided Biopsy OR pre-biopsy MRI OR (pre biopsy MRI) |
| #13 | #10 AND NOT (#11 OR #12) |
| #14 | Outcome Assessment, Health Care OR Benefits OR (Benefits and Costs) OR Harms OR Patient Harm OR Mortality OR Prostate Cancer-Specific Survival OR Prostate Cancer-Specific Death |
| #15 | Overdiagnosis OR Overtreatment |
| #16 | Quality of Life OR Emotions OR Decision Making |
| #17 | #14 OR #15 OR #16 |
| #18 | Europe OR United Kingdom OR UK OR Canada OR European OR British |
| #19 | Drugs OR Recurrence OR Active Surveillance OR Hormone |
| #20 | #3 AND #9 AND #13 AND #17 AND #18 AND NOT #19 |

***Supplementary Table 3.*** *Search terms and combinations used in Cochrane Central Register of Controlled Trials.*  The following selection filters to final search line: English language, clinical trials and randomised controlled trial studies, published between 01/01/2005 and 25/01/2023.

| #1 | Prostatic neoplasms OR Prostate cancer |
| --- | --- |
| #2 | Middle Aged OR Aged |
| #3 | #1 AND #2 |
| #4 | PSA OR Prostate-Specific Antigen / blood* |
| #5 | Mass Screening methods* OR Early Detection of Cancer |
| #6 | Magnetic Resonance Imaging OR Biparametric Magnetic Resonance Imaging OR Multiparametric Magnetic Resonance Imaging |
| #7 | Image-Guided Biopsy OR pre-biopsy MRI OR (pre biopsy MRI) |
| #8 | Risk Assessment* OR #Risk Stratification OR (Cambridge Prognostic group* OR CPG) OR (D'Amico OR Damico) |
| #9 | #4 AND (#5 OR #6 OR #7 OR #8) |
| #10 | PSA OR Prostate-Specific Antigen / blood* |
| #11 | Mass Screening methods* OR Early Detection of Cancer |
| #12 | Magnetic Resonance Imaging OR Biparametric Magnetic Resonance Imaging OR Multiparametric Magnetic Resonance Imaging |
| #13 | Image-Guided Biopsy OR pre-biopsy MRI OR (pre biopsy MRI) |
| #14 | #10 NOT (#11 OR #12 OR #13) |
| #15 | Outcome Assessment, Health Care OR Benefits OR (Benefits and Costs) OR Harms OR Patient Harm OR Mortality OR Prostate Cancer-Specific Survival OR Prostate Cancer-Specific Death |
| #16 | Overdiagnosis OR Overtreatment |
| #17 | Quality of Life OR Emotions OR Decision Making |
| #18 | #15 OR #16 OR #17 |
| #19 | Europe OR United Kingdom OR UK OR Canada OR European OR British |
| #20 | Drugs OR Recurrence OR Active Surveillance OR Hormone |
| #21 | #3 AND #9 AND #14 AND #18 AND #19 NOT #20 |

***Supplementary Table 4***. Proportion of areas providing mpMRI, 2019 Freedom of Information results.

| Region | Offering to PROMIS standard | Offering without DCE | Not offering at all |
| --- | --- | --- | --- |
| UK Wide | 72% (107/148) | 24% (35/148) | 4% (6/148) |
| England | 75% (91/122) | 23 (28/122) | 2% (3/122) |
| Scotland | 79% (11/14) | 7% (1/14) | 14% (2/14) |
| Wales | 57% (4/7) | 29% (2/7) | 14% (1/7) |
| Northern Ireland | 20% (1/5) | 80% (4/5) | 0% (0/5) |

Abbreviations: DCE, Dynamic Contrast-Enhanced; mpMRI, multiparametric magnetic resonance imaging; PROMIS, PROstate Magnetic resonance Imaging Study
